# Acute, subacute oral toxicity and Ames test of Py-mulin: an antibacterial drug candidate

**DOI:** 10.1186/s40360-021-00543-5

**Published:** 2022-01-04

**Authors:** Yuan Fan, Yunxing Fu, Yuhang Zhou, Yu Liu, Baocheng Hao, Ruofeng Shang

**Affiliations:** 1 Key Laboratory of New Animal Drug Project, Gansu Province/Key Laboratory of Veterinary Pharmaceutical Development, Ministry of Agriculture and Rural Affairs/Lanzhou Institute of Husbandry and Pharmaceutical Sciences of Chinese Academy of Agriculture Sciences, No. 335, Qilihe District, Lanzhou, 730050 People’s Republic of China; 2 Zhengzhou Key Laboratory of Immunopharmacology of effective components of Chinese Veterinary Medicine, College of Veterinary Medicine, Henan University of Animal Husbandry and Economy, 450046 Zhengzhou, People’s Republic of China

**Keywords:** Py-mulin, Acute toxicity, Subacute toxicity, Ames test

## Abstract

**Background:**

Py-mulin is a new pleuromutilin derivative with potent antibacterial activities in vitro and in vivo, suggesting this compound may lead to a promising antibacterial drug after further development. The present study is aimed to evaluate the acute and subacute oral toxicity, and the genotoxicity with the standard Ames test according to standard protocols.

**Methods:**

Acute oral toxicity of Py-mulin was determined using Kunming mice. The 28-day repeated dose oral toxicity study in SD rats was performed according to OECD guideline No. 407. The bacterial reverse mutation (Ames test) was carried out using four *Salmonella typhimurium* (*S. typhimurium*) strains TA97, TA98, TA100 and TA1535 with and without S9 metabolic activation.

**Results:**

The LD_50_ values in acute oral toxicity were 2973 mg/kg (female mice) and 3891 mg/kg (male mice) calculated by the Bliss method. In subacute toxicity study, 50 mg/kg Py-mulin did not induce any abnormality in body weight, food consumption, clinical sign, hematology, clinical chemistry, organ weight, and histopathology in all of the treatment groups. However, high doses of Py-mulin (100 and 300 mg/kg) displayed slightly hepatotoxicity to female rats. Furthermore, Py-mulin did not significantly increase the number of revertant colonies of four standard *S. typhimurium* strains with the doses of 0.16–1000 μg/plate in the Ames study.

**Conclusions:**

Based on our findings, our study provides some information for the safety profile of Py-mulin.

## Background

Bacteria with drug- resistance are a significant public health concern [1, 2]. Of particular concern are methicillin-resistant *Staphylococcus aureus* (MRSA) which caused more than 15,000 deaths in the U.S. each year [3]. Unfortunately, the spreading bacterial resistance makes most antibiotics lose efficiency [4]. Therefore, it is absolutely necessary to find and develop novel antimicrobial agents that do not produce cross resistance to antibiotics used in clinic [5].

In 1951, pleuromutilin was first discovered as a natural compound. Its derivatives selectively interact with prokaryotic ribosomes and thus inhibit bacterial protein synthesis [6–8]. This interaction mode was further confirmed by the crystallography data of 50S ribosomal subunit which were obtained from *Deinococcus radiodurans* in complex with tiamulin [9, 10].

After clarifying the structure of pleuromutilin in the 1960s, thousands of new derivatives were synthesized to improve it antimicrobial activity [11–13]. The modification of the pleuromutilin glycolic ester, especially the presence of thioether group, has been shown to give derivatives with improved antibacterial activities [14, 15], and thus has led to four drugs: tiamulin, valnemulin, retapamulin and lefamulin [16–20]. Tiamulin and valnemulin were approved as veterinary antibiotics for swine dysentery, enzootic pneumonia and chronic respiratory disease in poultry [16, 17, 21]. Retapamulin was for human topical skin infections [19]. Lefamulin was recently approved by FDA for treatment of community-acquired bacterial pneumonia (CABP) [20].

Py-mulin (14-O-[(4-Amino-6-hydroxy-pyrimidine-2-yl) thioacetyl] mutilin) is a new pleuromutilin derivative with a pyrimidine moiety. This compound displayed excellent antibacterial activity in vitro and in vivo against *Staphylococcus aureus* (*S. aureus*), MRSA and *Bacillus subtilis* (*B. subtilis*) [22]. Here we conducted acute and subacute oral toxicity and Ames test to provide data for demonstrating its safety.

## Methods

### Chemicals

Py-mulin (white powder) was synthesized in our lab and the synthetic pathway and confirmations of it structure by IR, NMR and HR-MS spectrometry were described previously [22]. The purity of Py-mulin was checked by HPLC analyses with 99.2%. During the study, the test sample was stored in the dark at a temperature of 4 °C and dissolved in DMSO freshly before use. All the other chemicals and solvents used were obtained from standard vendors and used without further purification.

### Animals

Adult specific pathogen free (SPF) Kunming mice (4–5 weeks old) and Sprague-Dawley (SD) rats (4–6 weeks old) were purchased from the Laboratory Animal Center of Lanzhou University (Lanzhou, China). The animals were separated by sex, housed in clean stainless steel cages (two rat per cage, three mice per cage), free access to food and water, and maintained under the 23 °C conditions with a constant 12 h light-dark cycle. Animals were acclimated for at least 7 days prior to the experiment. All experiments were carried out between 08:30 AM and 17:30 PM. The experimental procedures were performed in accordance with the Ethical Principles in Animal Research and were approved by the Committee for Ethics in the Laboratory Animal Center of Lanzhou University (number: SCXK2013–0002). The study was carried out in compliance with the ARRIVE guidelines.

### Acute Oral toxicity in mice

After being stratified by weight, the SPF mice (30 male and 30 female, initial weight of 20.2–24.1 g and 19.3–21.8 g, respectively) were randomly assigned to six groups, including five treatment groups and one vehicle control group (5 female and 5 male mice for each group). The vehicle control group received only vehicle (ethyl oleate:1,2-propanediol:tween-80: sterile water = 4:10:31:55) in a volume of 10 mL/kg body weight (b.w.) by oral gavage. After mice were fasted for 3 h, Py-mulin was dissolved in vehicle and orally administered to the mice at doses of 55, 175, 550, 1750 and 5000 mg/kg b.w. (with volume of 10 mL/kg), respectively. Vehicle or Py-mulin was administered only once (on day 0) and animals were observed for next 14-days post treatment. All mice were observed twice daily for behavioural symptoms and mortality for two week. Individual body weights were recorded on days 0, 7 and 14. All of the surviving animals were euthanized by anesthetized with pentobarbital sodium 0.06 g/kg (i.p.) at the end of the observation period of 14 days, and their vital organs were individually observed for overt pathology by necropsy. Finally, the LD_50_ and 95% confidence interval for female and male mice were calculated by the probit weighted regression method (Bliss method) using IBM SPSS Statistics for Windows version 24.0 (SPSS Inc., Chicago, USA).

### Subacute toxicity

#### Study design

The subacute toxicity study of Py-mulin was performed according to OECD guideline No. 407 [23]. Female and male SD rats (initial weight of 126.3–145.6 g and 141.2–158.1 g, respectively) were randomly divided into four groups after one week of acclimatization. Each group consisted of 10 female rats and 10 male rats. Doses were determined based on the result of acute oral toxicity. Py-mulin was dissolved in vehicle (ethyl oleate:1,2-propanediol:tween-80: sterile water = 4:10:31:55) and administrated to rats by oral gavage at doses of 50, 100 and 300 mg/kg bw or control (4 mL/kg bw vehicle) for 28 days (administration at the same time each day ±1 h). All the animals were observed once daily to detect toxic symptoms. During the whole experiment, body weight and food consumption were measured every week and the gavage volume was adjusted based on the last measured body weight.

#### Clinical observations

During the 28-day treatment, for all the rats, behavioral changes, symptoms and signs of gross toxicity, food consumption, water intake and mortality were closely observed at least once per day. The individual body weights of the rats and food consumption were measured weekly. The mean weekly body weight gain and daily food consumption were calculated for each sex animal and dose level.

#### Haematological and biochemical analysis

At the end of the experiment, following an overnight fast, rats were anesthetized with excess of CO_2_ and euthanized by exsanguination. Blood sample (5 mL) of animal in each group were collected from the femoral artery in ethylenediaminetetraacetic acid (EDTA) coated tubes and non-heparinized tubes for the haematological and biochemical analysis, respectively [24]. The haematological parameters, including the white blood cell (WBC), the hemoglobin (HGB), red blood cell (RBC) counts, hematocrit (HCT) levels, mean corpuscular hemoglobin (MCH), mean corpuscular volume (MCV), mean corpuscular haemoglobin concentration (MCHC) and number of platelets (PLT), were determined using a Poche-100iv Diff instrument (SYSMEX, Kakogawa, Japan). The biochemical parameters measured were alanine transaminase (ALT), aspartate transaminase (AST), alkaline phosphatase (ALP), total bilirubin (TB), total cholesterol (TC), low density lipoprotein (LDL), high density lipoprotein (HDL), triglycerides (TG), urea nitrogen (UN), creatinine (Crea), total protein (TP), glucose (Glu), albumin (Alb), calcium (Ca) and phosphorous (P), using reagent kits and a Mindray BS-420 auto hematology analyzer (Mindray Corporation, Shenzhen, China).

#### Macroscopic examination and organ weights

After necropsy, each rat was examined for the external surface, visceral organs and all orifices, followed by recording the gross pathological changes. Finally, the heart, liver, spleen, lungs, kidneys, thymus, testes or ovaries were collected and weighed. The relative organ weight (organ/body-weight ratio) was calculated based on the final animal’s body weight.

#### Histopathologic examination

Histopathological examinations were performed on the kidneys, spleen, heart, liver, thymus, lungs, small intestines, testes or ovaries of randomly selected five female and five male rats using a routine paraffin-embedding technique. The organs were preserved in 10% neutral-buffered formalin, and their slides were prepared. Upon completion of staining with hematoxylin and eosin (H&E), microscopic examinations were performed on all the mentioned tissues.

### Ames test

*S. typhimurium* strains, including TA97, TA98, TA100 and TA1535, were first checked for their genetic integrity. Biotin dependence, *rfa* marker (crystal violet), biotin and histidine dependence, histidine dependence and presence of the plasmid pKM101 (ampicillin resistance) or pAQ1 (tetracycline resistance) were then tested. Different strains were used to identify different types of mutations.

The test was performed with five concentrations of Py-mulin. After weighing accurately, 100 mg Py-mulin was dissolved in DMSO (2 mL) and the obtained solution (1 mL) was diluted by fivefold with distilled water. The 0.5 mL obtained serial dilutions were diluted with 9.5 mL melted top agar to the desired concentrations (500, 100, 20, 4 and 0.8 μg/mL). DMSO (0.5 mL) was directly incorporated into 9.5 mL melted top agar (the final concentrations was 100 μL/plate) as the vehicle control. For all bacterial strains, 2-aminofluorene and fenaminosulf (the final concentration of 10 μL/plate) were used as positive control with and without S9, respectively. As described in the literatures [25, 26], the Ames test was conducted with some modifications. Each strain (1 mL) was inoculated into 5 mL of nutrient broth, followed by incubating overnight at 37 °C. The mixture comprised respective strains, DMSO, 2-aminofluorene and drug for testing with the S9. For testing without S9, 1 × phosphate buffer saline (PBS, pH 7.4) replaced the S9 mix. The obtained mixtures were then added into the tube, mixed well and incubated (20 min at 37 °C). Then, the top agar (2 mL) was melted and added to each tube and gently mixed, followed by pouring onto the surface of glucose minimal (GM) agar plate (the final concentrations of Py-mulin were 1000, 200, 40, 8 and 1.6 μg/plate, respectively, and the final concentrations of DMSO was 100 μL/plate). The plate was then swirled to distribute the overlay agar to all surfaces of GM agar, followed by inverting and incubation (37 °C for 48 h) when the top agar solidified. The resultant colonies were counted manually as the number of revertant colonies per plate. The experiments were performed in triplicate.

### Statistical analysis

We used IBM SPSS Statistics for Windows version 24.0 [27] to perform the statistical analysis. Using One-way analysis of variance (ANOVA) and Dunnett’s *post-hoc* tests as appropriate analyzed the data. Statistical significant difference was defined as a *p* < 0.05 and the extremely significant difference was defined as a *p* < 0.01.

## Results

### Acute oral toxicity

**Fig. 1:**
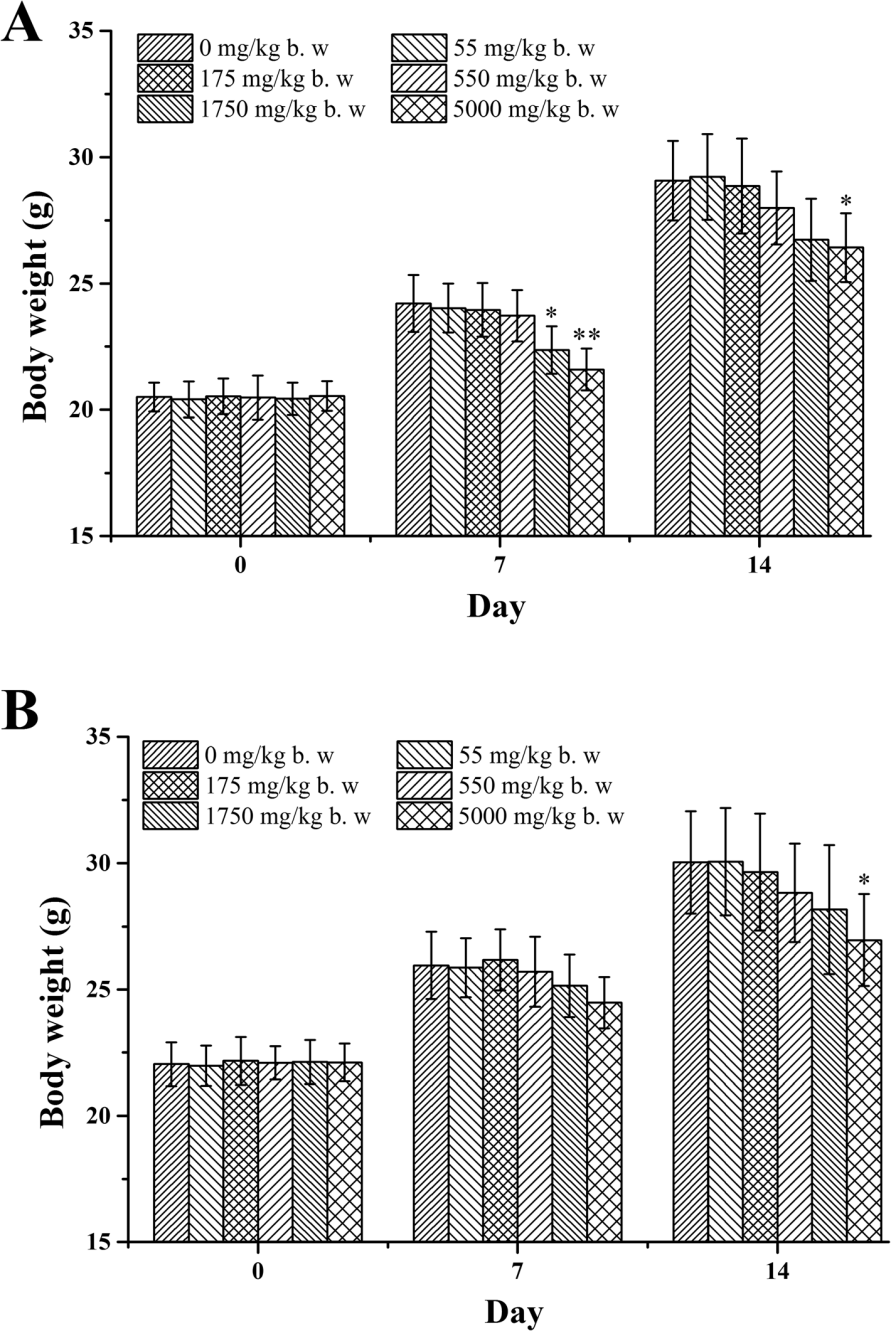
Body weight gain of female (**A**) and male (**B**) mice administered with Py-mulin. The results are expressed as mean ± SD. *p < 0.05 versus 0 mg/kg b. w. group

No animals died after receiving an oral dose of 55, 175 and 550 mg/kg of Py-mulin. Conversely, Py-mulin caused 20 and 70% mortality which were found in both female and male animals at the dose of 1750 (one female and one male mice died) and 5000 mg/kg (four female and three male mice died) within three days, respectively. Using the Bliss method, the LD_50_ values were determined to be 2973 and 3891 mg/kg in female and male mice, with the confidence level of 95% was 975 to 14,445 mg/kg and 1474 to 0.5599 mg/kg, respectively. No clinical signs or adverse effects of toxicity were observed for the surviving animals receiving the dose of 55, 175 and 550 mg/kg of Py-mulin during the study. Clinical signs, including lethargy, rough coat, prostration, squinting, gait disturbance and bradykinesia, of acute toxicity occurred in mice given single oral doses of 1750 and 5000 mg/kg, appearing approximately 8 to 12 h after dosing. In female and male mice treated with Py-mulin, body weight generally increased throughout the study for all dose groups (Fig. 1). Compared to the control, body weight of female mice was reduced by 7.64 and 10.82% at day 7 in the 1750 and 5000 mg/kg groups (*p* < 0.05 and *p* < 0.01, respectively). In the 5000 mg/kg group, compared to the control at day 14, body weight of female and male mice was reduced 9.12 and 10.22% (*p* < 0.05), respectively. At necropsy, no gross pathological abnormalities were found in any organs of the surviving animals receiving the dose of 55, 175 and 550 mg/kg of Py-mulin. However, hemorrhage on the lungs, livers and small intestines were found in died mice during the treatment or some mice in higher dose groups (1750 and 5000 mg/kg group). Furthermore, drug precipitation was also found in the stomachs of some died mice at the early days which were given high dose of 5000 mg/kg of Py-mulin.

### Subacute 28 days study

#### Observational study, body weights, food consumption and water intake

**Fig. 2:**
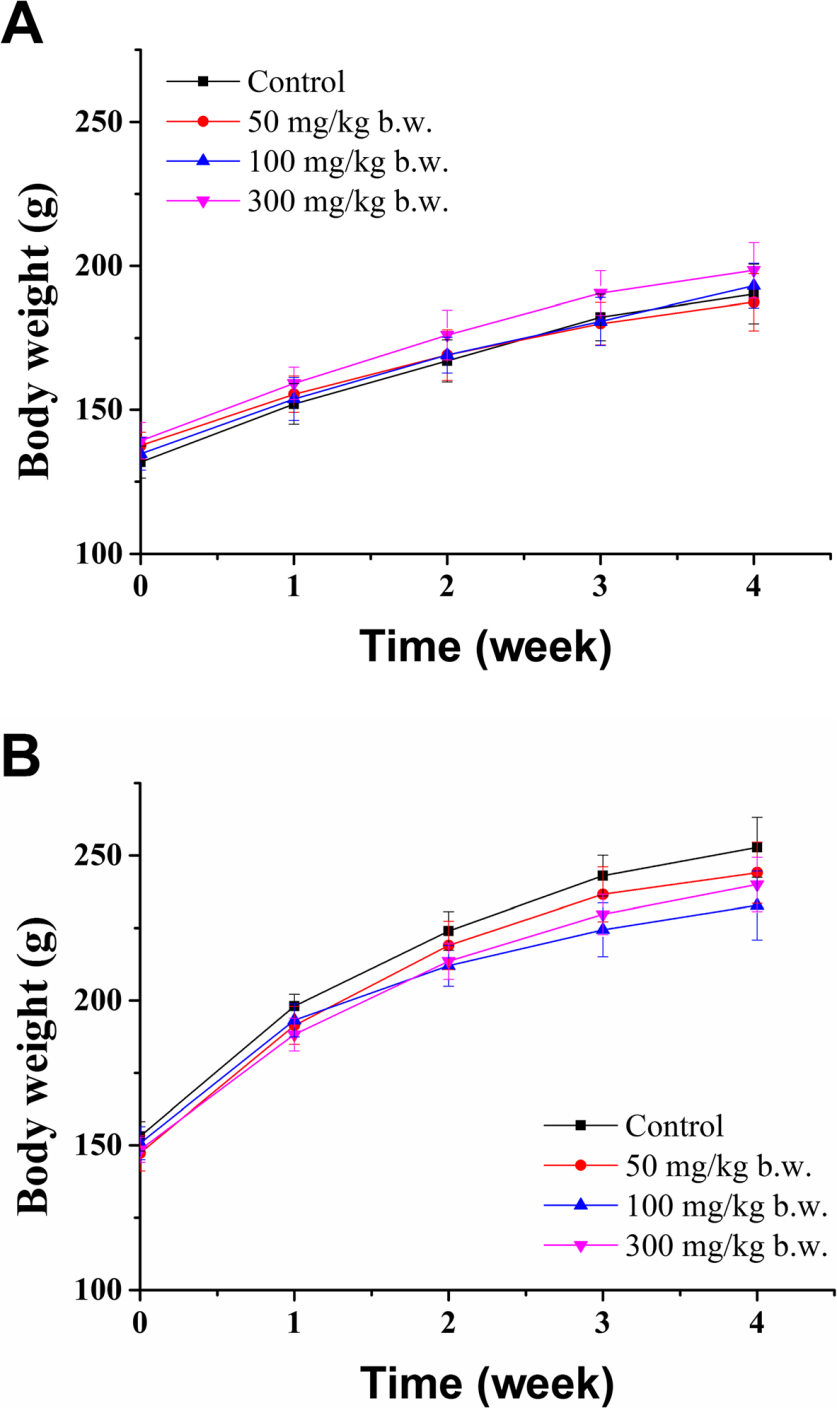
Body weight of female (**A**) and male (**B**) rats in subacute toxicity study of Py-mulin (*n* = 10)

No deaths or obvious clinical signs related to toxicity were observed in any treated groups or the vehicle control group throughout the 28-day experimental period. The body weights of animals are shown in Fig. 2. Food and water consumption for all rats in control and treated groups were normal and similar at any time point.

#### Haematology and clinical biochemistry

Hematological values of rats treated orally with Py-mulin for 28 days are shown in Table 1. Compared with the negative control, RBC in females and MCV in males significantly decreased in 50 mg/kg dose of Py-mulin treated groups (*p* < 0.05), while HCT in females (300 mg/kg) significantly increased (*p* < 0.05). However, for the other haematological parameters, no significant difference was observed between the treated groups and the control group.

**Table 1:** Haematology parameters of rats sacrificed on day 28 of 28-day feeding test (Mean ± SD)

Parameters	0 mg/kg b. w.	50 mg/kg b. w.	100 mg/kg b. w.	300 mg/kg b. w.
Female				
WBC	5.47 ± 1.11	4.94 ± 1.33	4.16 ± 1.08	4.83 ± 1.48
RBC	8.33 ± 0.98	6.82 ± 1.01*	7.85 ± 0.50	7.50 ± 0.53
HGB	141.42 ± 8.77	131.37 ± 7.91	135.16 ± 5.94	139.01 ± 7.24
HCT	40.96 ± 1.66	43.88 ± 2.51	41.24 ± 1.61	44.01 ± 1.87*
MCV	57.21 ± 1.32	58.56 ± 1.08	56.73 ± 1.43	56.84 ± 1.22
MCH	21.60 ± 0.79	22.07 ± 0.95	20.81 ± 0.98	21.13 ± 0.88
MCHC	357.47 ± 12.04	360.10 ± 10.35	348.17 ± 19.45	350.26 ± 13.55
PLT	1011.28 ± 124.25	911.31 ± 118.19	1180.69 ± 158.54	915.68 ± 135.97
Male				
WBC	7.34 ± 1.56	8.21 ± 1.73	6.90 ± 2.11	7.67 ± 1.26
RBC	8.37 ± 0.67	8.48 ± 0.88	7.88 ± 0.72	8.73 ± 0.90
HGB	145.84 ± 7.90	139.36 ± 8.40	138.26 ± 6.99	143.52 ± 9.31
HCT	40.72 ± 1.37	40.22 ± 1.83	41.91 ± 1.60	39.86 ± 2.51
MCV	57.75 ± 1.30	55.98 ± 0.98*	58.24 ± 1.31	59.03 ± 1.04
MCH	21.67 ± 0.87	20.89 ± 0.93	21.32 ± 0.75	21.56 ± 0.87
MCHC	341.83 ± 15.83	352.21 ± 21.01	354.37 ± 11.90	360.81 ± 19.39
PLT	952.67 ± 142.45	893.28 ± 107.30	995.37 ± 135.60	979.47 ± 150.94

Abbreviations: *WBC* white blood cells (10^9^/L), *RBC* red blood cells (10^12^/L), *HGB* hemoglobin (g/L), *HCT* hematocrit (%), *MCV* mean corpuscular volume, *MCH* mean corpuscular hemoglobin, *MCHC* mean corpuscular hemoglobin concentration, *PLT* platelets (10^3^/μL). Values represent means ± SD. *p < 0.05 vs. control group (0 mg/kg b. w.)

After administration of Py-mulin, significant changes were also observed in clinical chemistry analyses (Table 2), included increased ALT in females (100 and 300 mg/kg, *p* < 0.01 and 0.05, respectively), decreased TB in female (50 mg/kg, *p* < 0.05), increased HDL in females (100 mg/kg, *p* < 0.05), decreased UN in female (100 mg/kg, *p* < 0.05), increased ALP in male (100 mg/kg, *p* < 0.05) and decreased P in males (300 mg/kg, *p* < 0.05).

**Table 2:** Biochemical parameters of rats sacrificed on day 28 of 28-day feeding test (Mean ± SD)

Parameters	0 mg/kg b. w.	50 mg/kg b. w.	100 mg/kg b. w.	300 mg/kg b. w.
Female				
ALT (U/L)	44.01 ± 5.89	45.64 ± 6.73	59.08 ± 6.99**	51.78 ± 4.82*
ALP (U/L)	243.86 ± 36.02	196.54 ± 42.84	220.15 ± 45.12	198.22 ± 36.86
AST (U/L)	117.47 ± 23.55	108.93 ± 26.11	122.98 ± 24.20	118.80 ± 23.07
TB (μmol/L)	2.91 ± 0.40	2.25 ± 0. 37*	3.08 ± 0.43	2.34 ± 0.47
TC (mmol/L)	1.54 ± 0.23	1.57 ± 0.19	1.70 ± 0.18	1.41 ± 0.15
HDL (mmol/L)	0.47 ± 0.05	0.54 ± 0.06	0.57 ± 0.08*	0.45 ± 0.07
LDL (mmol/L)	0.78 ± 0.08	0.80 ± 0.06	0.86 ± 0.06	0.79 ± 0.06
TG (mmol/L)	0.67 ± 0.34	0.74 ± 0.40	0.82 ± 0.39	0.71 ± 0.27
Crea (μmol/L)	39.65 ± 6.73	38.08 ± 5.78	36.76 ± 6.78	40.29 ± 6.26
UN (mmol/L)	6.97 ± 0.67	7.09 ± 0.77	5.89 ± 0.68*	6.74 ± 0.47
TP (g/L)	64.77 ± 4.99	60.92 ± 5.38	65.69 ± 5.50	58.89 ± 4.69
Alb (g/L)	31.33 ± 3.39	31.17 ± 3.03	30.28 ± 4.53	30.03 ± 3.68
Glu (mmol/L)	5.62 ± 0.68	6.18 ± 0.50	6.15 ± 0.45	5.59 ± 0.59
Ca (mmol/L)	2.64 ± 0.50	2.39 ± 0.38	2.56 ± 0.52	2.38 ± 0.46
P (mmol/L)	3.00 ± 0.25	2.64 ± 0.31	2.79 ± 0.30	2.92 ± 0.27
Male				
ALT (U/L)	52.45 ± 5.76	60.28 ± 6.72	47.93 ± 5.46	51.42 ± 6.49
ALP (U/L)	246.66 ± 42.88	257.62 ± 37.88	283.76 ± 46.69*	222.44 ± 40.11
AST (U/L)	110.45 ± 24.35	116.05 ± 20.23	125.27 ± 26.17	93.63 ± 24.24
TB (μmol/L)	2.64 ± 0.37	2.93 ± 0.54	2.64 ± 0.41	2.10 ± 0.46
TC (mmol/L)	1.54 ± 0.16	1.55 ± 0.17	1.61 ± 0.21	1.44 ± 0.23
HDL (mmol/L)	0.52 ± 0.08	0.50 ± 0.05	0.57 ± 0.09	0.54 ± 0.08
LDL (mmol/L)	0.85 ± 0.08	0.88 ± 0.06	0.85 ± 0.04	0.80 ± 0.07
TG (mmol/L)	0.69 ± 0.38	0.75 ± 0.40	0.86 ± 0.33	0.59 ± 0.30
Crea (μmol/L)	36.00 ± 4.61	42.27 ± 6.47	35.52 ± 5.65	30.79 ± 6.13
UN (mmol/L)	5.42 ± 0.43	5.16 ± 0.49	6.13 ± 0.76	5.08 ± 0.56
TP (g/L)	62.83 ± 4.85	69.64 ± 5.80	68.36 ± 4.76	64.29 ± 4.33
Alb (g/L)	30.20 ± 4.02	31.18 ± 4.08	30.90 ± 3.55	28.49 ± 3.15
Glu (mmol/L)	4.24 ± 0.55	4.92 ± 0.56	4.04 ± 0.49	3.83 ± 0.52
Ca (mmol/L)	2.34 ± 0.38	2.53 ± 0.51	2.50 ± 0.45	1.97 ± 0.38
P (mmol/L)	2.83 ± 0.21	2.68 ± 0.27	3.05 ± 0.25	2.44 ± 0.27*

Abbreviations: *ALT* Alanine transaminase, *ALP* Alkaline phosphatase, *AST* Aspartate transaminase, *TB* Total bilirubin, *TC* Total cholesterol, *HDL* High density lipoprotein, *LDL* low density lipoprotein, *TG* triglycerides, *Crea* Creatinine, *UN* Urea nitrogen, *TP* Total protein, *Alb* Albumin, *Glu* Glucose, *Ca* Calcium, *P* phosphorous. Values represent means ± SD. *p < 0.05, **p < 0.01 vs. control group (0 mg/kg b. w.)

#### Macroscopic observation, absolute organ weight and relative organ weights

There was no macroscopic pathological change in Py-mulin treated groups on the 28th day. The organ weight and organ/body-weight ratios of female and male rats were summarized in Table 3 and Table 4. In female rats, there were significant increases (*p* < 0.05) in the absolute organ weight of the liver (300 mg/kg), and significantly increased (*p* < 0.05) in the relative weight of the liver (300 mg/kg) and the thymus (100 mg/kg) compared to that of the control group. In male rats, absolute thymus (100 mg/kg) weight was significantly decreased (*p* < 0.05) and the relative weight of testis (100 mg/kg) significantly increased (*p* < 0.05) compared to that of control group.

**Table 3:** Absolute and relative organ weights from female rats treated orally with Py-mulin for 28 days

Parameters	0 mg/kg b.w.	50 mg/kg b.w.	100 mg/kg b.w.	300 mg/kg b.w.
Absolute organ weight (g)				
Heart	0.75 ± 0.10	0.70 ± 0.10	0.82 ± 0.06	0.81 ± 0.07
Liver	6.89 ± 0.83	7.29 ± 0.90	7.65 ± 1.01	7.88 ± 0.53*
Spleen	0.44 ± 0.08	0.48 ± 0.05	0.42 ± 0.08	0.49 ± 0.07
Lungs	1.04 ± 0.11	1.12 ± 0.13	1.08 ± 0.12	1.05 ± 0.14
Kidneys	1.35 ± 0.14	1.39 ± 0.17	1.41 ± 0.15	1.43 ± 0.17
Thymus	0.35 ± 0.05	0.37 ± 0.06	0.42 ± 0.06	0.41 ± 0.07
Ovaries	0.09 ± 0.02	0.12 ± 0.03	0.11 ± 0.03	0.11 ± 0.04
Organ-to-body weight ratio (%)				
Heart	0.39 ± 0.04	0.37 ± 0.04	0.42 ± 0.03	0.41 ± 0.03
Liver	3.61 ± 0.30	3.88 ± 0.33	3.95 ± 0.40	3.97 ± 0.10*
Spleen	0.23 ± 0.03	0.26 ± 0.03	0.22 ± 0.03	0.25 ± 0.03
Lungs	0. 55 ± 0.05	0.59 ± 0.04	0.56 ± 0.04	0.52 ± 0.06
Kidneys	0.71 ± 0.05	0.74 ± 0.06	0.73 ± 0.07	0.72 ± 0.05
Thymus	0.18 ± 0.02	0.20 ± 0.03	0.22 ± 0.02*	0.21 ± 0.03
Ovaries	0.05 ± 0.01	0.06 ± 0.02	0.06 ± 0.02	0.06 ± 0.02

Values are mean ± SD for 10 rats in each group

*Statistically significant difference compared to control (*p* < 0.05)

**Table 4:** Absolute and relative organ weights from male rats treated orally with Py-mulin for 28 days

Parameters	0 mg/kg b.w.	50 mg/kg b.w.	100 mg/kg b.w.	300 mg/kg b.w.
Absolute organ weight (g)				
Heart	0.94 ± 0.0.09	0.93 ± 0.08	0.88 ± 0.13	0.97 ± 0.12
Liver	9.82 ± 0.76	9.55 ± 0.53	9.08 ± 0.68	9.69 ± 0.40
Spleen	0.58 ± 0.10	0.54 ± 0.11	0.56 ± 0.08	0.52 ± 0.07
Lungs	1.31 ± 0.21	1.25 ± 0.16	1.10 ± 0.12	1.17 ± 0.13
Kidneys	1.89 ± 0.22	1.82 ± 0.17	1.75 ± 0.19	1.85 ± 0.28
Thymus	0.44 ± 0.07	0.45 ± 0.12	0.34 ± 0.06*	0.47 ± 0.11
Testis	2.69 ± 0.22	2.68 ± 0.18	2.67 ± 0.18	2.72 ± 0.23
relative organ weight				
Heart	0.37 ± 0.03	0.38 ± 0.03	0.38 ± 0.04	0.40 ± 0.04
Liver	3.88 ± 0.24	3.91 ± 0.12	3.90 ± 0.14	4.04 ± 0.12
Spleen	0.23 ± 0.03	0.22 ± 0.04	0.24 ± 0.03	0.22 ± 0.02
Lungs	0.52 ± 0.06	0.51 ± 0.06	0.47 ± 0.03	0.493 ± 0.04
Kidneys	0.74 ± 0.06	0.75 ± 0.04	0.75 ± 0.08	0.77 ± 0.09
Thymus	0.17 ± 0.02	0.18 ± 0.04	0.15 ± 0.03	0.19 ± 0.04
Testis	1.06 ± 0.05	1.10 ± 0.07	1.15 ± 0.06*	1.13 ± 0.06

Values are mean ± SD for 10 rats in each group

*Statistically significant difference compared to control (*p* < 0.05)

#### Histopathological analysis

**Fig. 3:**
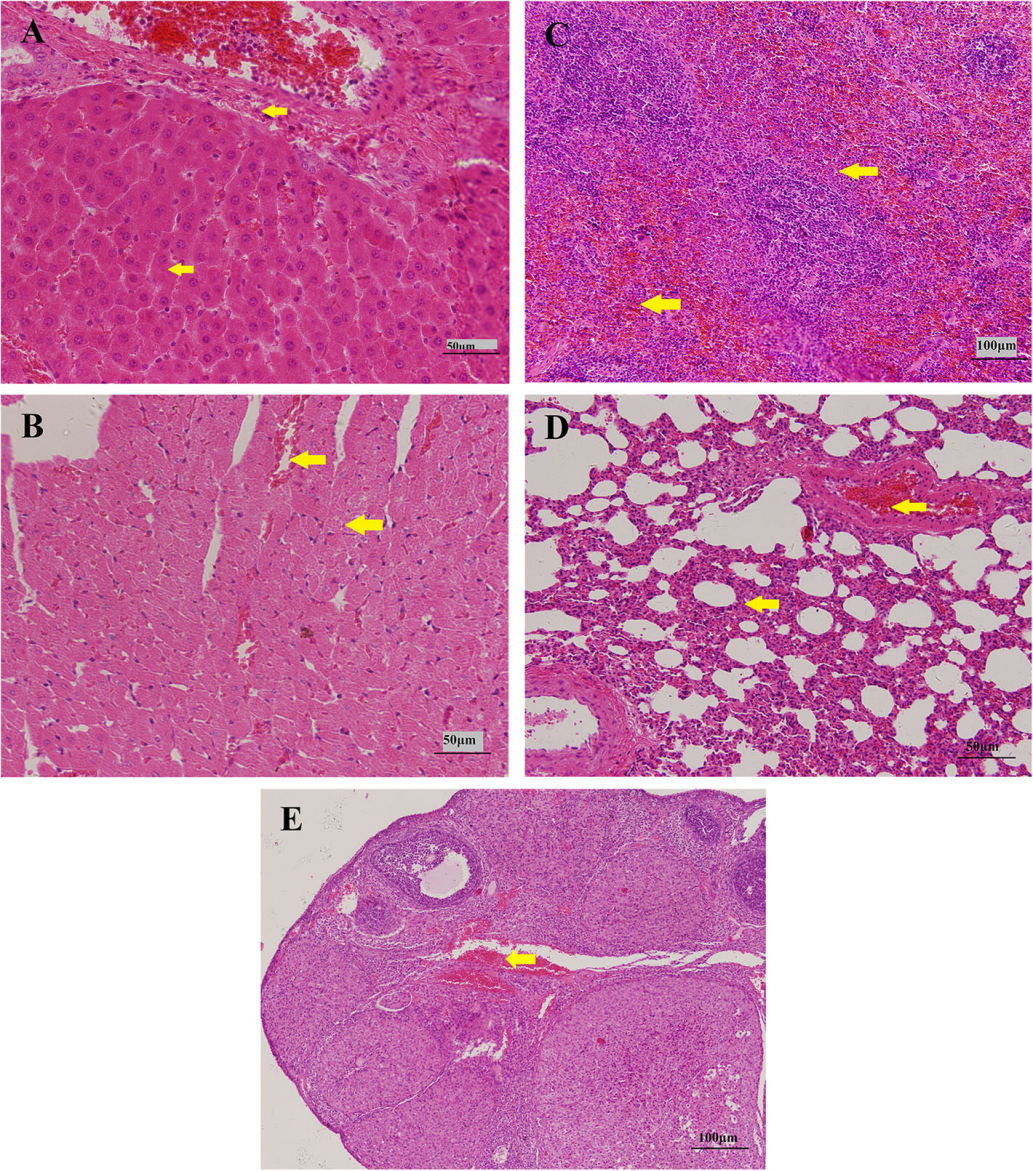
Selected microphotographs (H&E × 100) of tissues in rats during 28 days repeated dose toxicity study. **A**: liver (300 mg/kg b.w., female), inflammatory cells and edema; **B**: heart (100 mg/kg b.w., female), erythrocytosis and edema; **C**: spleen (100 mg/kg b.w., male), erythrocytosis and congestion; **D** lung (50 mg/kg b.w., female), connective tissues proliferation and congestion; **E**: ovaries (100 mg/kg b.w., female), congestion

Treatment-related changes in rat heart, liver, spleen, lungs, kidneys, thymus, small intestines, testes or ovaries were confirmed by H&E histology analyses in the three dose groups of Py-mulin as compared to the control group. Evaluation of kidneys, thymus, testes and small intestines did not reveal any histopathological changes. Typically histopathological characters of liver, heart, spleen, lung and ovaries in control and the three treated groups were showed in Fig. 3 and Table 5. Various lesions, including mild congestion, inflammatory cells and edema in the liver (Fig. 3A), erythrocytosis and edema in heart (Fig. 3B), erythrocytosis and congestion in spleen (Fig. 3C), connective tissues proliferation and congestion in lungs (Fig. 3D) and congestion in ovaries (Fig. 3E) were observed in three treatment groups and control group. However, the severities of all of these lesions were minimal or slight.

**Table 5:** Histopathology of rats on day 28 of subacute feeding test of Py-mulin

Organ	Histopathological characters	28-day (n = 10)							
		Control		50 mg/kg b.w.		100 mg/kg b.w.		300 mg/kg b.w.	
		Female	Male	Female	Male	Female	Male	Female	Male
Liver	Inflammatory cells	1/5	1/5	2/5	0/5	2/5	1/5	1/5	1/5
	Congestion	1/5	0/5	0/5	0/5	1/5	1/5	1/5	2/5
	Edema	0/5	0/5	0/5	1/5	1/5	0/5	2/5	1/5
Heart	Erythrocytosis	1/5	0/5	2/5	0/5	2/5	1/5	0/5	1/5
	Edema	0/5	0/5	1/5	0/5	3/5	0/5	1/5	0/5
Spleen	Erythrocytosis	0/5	1/5	0/5	1/5	1/5	1/5	1/5	1/5
	Congestion	0/5	0/5	1/5	0/5	1/5	1/5	0/5	1/5
Lungs	Connective tissues proliferation	1/5	1/5	1/5	2/5	1/5	0/5	2/5	2/5
	Congestion	0/5	1/5	1/5	0/5	0/5	0/5	0/5	2/5
Ovaries	Congestion	0/5	˗˗	2/5	˗˗	1/5	˗˗	1/5	˗˗

### Ames test

The results of the *S. typhimurium* reverse mutation assay for Py-mulin in the presence or absence of metabolic activation are shown in Table 6. Revertant colonies in all positive groups were much more than two-fold of colonies induced by five concentrations of Py-mulin and vehicle control (100 μL/plate DMSO). Py-mulin at 1000 μg/plate significantly decreased the number of revertant colonies of TA97 (−S9 and + S9) and TA100 (−S9 and + S9) due to its antibacterial property. At 200 μg/plate, Py-mulin also significantly decreased the number of revertant colonies of TA97 (+S9) and TA100 (−S9 and + S9). However, no significantly different between the number of revertant colonies induced by the vehicle control and Py-mulin at the 40, 8 or 1.6 μg/plate was observed.

**Table 6:** Ames test results of Py-mulin using *S. typhimurium* strains TA97, TA98, TA100, TA102 and TA1535

Substance	Dose (μg/plate)	TA97		TA98		TA100		TA1535	
		- S9	+ S9	- S9	+ S9	- S9	+ S9	- S9	+ S9
Py-mulin	1000	46.3 ± 3.5**	55.0 ± 4.6**	21.0 ± 2.6	19.7 ± 3.5	36.7 ± 3.1**	38.0 ± 3.6**	20.7 ± 3.1	27.3 ± 2.5
	200	62.0 ± 5.3	62.7 ± 4.2**	22.7 ± 3.1	26.7 ± 2.5	83.7 ± 4.5*	73.3 ± 3.1**	25.0 ± 2.6	38.7 ± 3.5
	40	72.0 ± 3.6	77.7 ± 3.2	33.3 ± 4.5	28.0 ± 4.9	93.7 ± 4.2	89.0 ± 8.5	34.3 ± 4.0	41.7 ± 4.7
	8	80.3 ± 4.7	79.3 ± 5.1	23.7 ± 4.0	24.7 ± 5.5	105.7 ± 6.7	101.3 ± 3.5	38.7 ± 4.7	36.7 ± 5.0
	1.6	76.3 ± 5.6	94.3 ± 4.0	32.7 ± 3.5	31.7 ± 3.1	112.0 ± 5.3	115.0 ± 5.0	42.0 ± 4.4	44.3 ± 5.5
Vehicle control		84.0 ± 5.0	98.3 ± 5.1	24.7 ± 3.5	29.7 ± 2.1	111.3 ± 4.2	120.0 ± 5.6	34.7 ± 4.2	43.3 ± 5.7
Positive control		737.3 ± 13.3**	964.7 ± 13.0**	567.7 ± 11.5**	614.0 ± 15.1**	913.3 ± 20.8**	1212.3 ± 12.9**	583.0 ± 13.7**	746.3 ± 12.6**

Note: The numbers indicate the means and standards deviation values of CFU in triplicate assay systems

+ S9: with metabolic activation; − S9: without metabolic activation

Vehicle contro: DMSO (100 μL/plate)

Positive control: 2-aminofluorene for all strains with S9; Fenaminosulf for all strains without S9

*p < 0.05, **p < 0.01 vs. vehicle control

## Discussion

Toxicity assessment is an essential process during the development and application of a new drug. This study evaluated the acute, subacute oral toxicity and genotoxicity of Py-mulin, a novel pleuromutilin derivative with a substituted pyrimidine moiety.

In the acute phase of the study, exposure of mice to a single dose of 55, 175 and 550 mg/kg did not produce any mortality and alterations in adverse clinical signs. However, a single dose of 1750 and 5000 mg/kg of Py-mulin showed clinical signs of acute toxicity and 20 and 70% mortality on the 3th day of post-dosing, respectively. The calculated LD_50_ values for Py-mulin were 2973 mg/kg (female mice) and 3891 mg/kg (male mice) which were all significant higher than that of tiamulin (1580 mg/kg in mice), and valnemulin (1710 mg/kg in mice), two pleuromutilin drugs used for veterinary [28, 29]. This reflected the low acute toxicity of Py-mulin in comparison with the same class drugs.

The 28-day subacute oral toxicity study of Py-mulin in rats showed similar mean body weights among the four groups during the experiment. Furthermore, this study revealed no test substance-related effects on body weights, food consumption or clinical signs, in any rats in the treatment groups up to 300 mg/kg b. w.

Hematological levels which are measured in the animal studies can be used to assess the risk of toxicity for human [24, 30]. All animals in the treated groups did not show any significant change when compared to that of the control. Only a few significant changes in hematological values (RBC, HCT and MCV) were observed in both female and male animals, but they were fluctuated within the physiological norm. The increase levels of AST ALP and ALT in the blood are associated with damage of hepatic cells [31]. The administration of Py-mulin in this subacute study caused a significant increase in the levels of ALT in female rats at 100 and 300 mg/kg dose. The levels of ALP also increased significantly in the male rats but decreased slightly in the female rats. Furthermore, the elevated levels of triglycerides, bilirubin and cholesterol and reduction in albumin are other significant indicators of failure in hepatic function [32, 33]. But in our study, the repeated administration of Py-mulin did not show any significant change in the level of triglycerides, cholesterol and albumin. In general, the hematological and biochemical changes were not dose-dependent.

At necropsy, the increase of both absolute and relative weight in the female rate liver was dose-dependent. Statistically significant differences were further observed in the 300 mg/kg female group. Furthermore, our further histopathological study showed the lesions in the liver, heart, spleen, lung and ovaries were sporadically detected in controls and the treated rats. But these observed histopathological changes were more in female than that in male rats in treatment groups. Combined with the result that Py-mulin with 100 and 300 mg/kg doses caused significant increase of ALT in female rats, it could be concluded that Py-mulin display slightly hepatotoxicity to female rats with 100 and 300 mg/kg doses.

For evaluating the genotoxic activity of a chemical agent, the Ames test is an initial in vitro screening method with rapid and effective properties [34, 35]. Four *S. typhimurium* strains TA97, TA98, TA100 and TA1535 with and without S9 metabolic activation were employed in the present study. The sensitivity of our Ames test was demonstrated by the facts that the chemicals in positive control group significantly increased the counts of corresponding mutant strain than that in vehicle control group. Due to its antibacterial property, the higher concentrations of Py-mulin decreased the number of revertant colonies, especially the TA97 and TA100 strains. In the presence and absence of S9, exposure to Py-mulin at 40, 8 and 1.6 μg/plate, respectively, did not display any inhibitory effect on the growth of *S. typhimurium*, indicating that the four strains were tolerant to these concentrations. Moreover, Py-mulin with all tested concentrations did not increase the counts of revertant colonies significantly in four strains with or without S9 activation.

## Conclusions

In summary, the acute toxicity study showed no acute toxicity for Kunming mice after the oral administration of Py-mulin up to 550 mg/kg, but indicated the acute oral lethal dose was more than 1750 mg/kg with an approximate LD_50_ of 2973 mg/kg (female mice) and 3891 mg/kg (male mice). The subacute oral toxicity study suggested that no biologically significant differences were observed in hematology, clinical chemistry, organ weights, or histopathology between 50 mg/kg treatment group and the control group. However, in female rats, hepatotoxicity was more likely to occur than the other examined organs following higher dose of Py-mulin (100 and 300 mg/kg). Ames test revealed that Py-mulin at concentrations of 1.6–1000 μg/plate did not induce gene mutation in the four standard *S. typhimurium* strains.

## Data Availability

The datasets supporting the conclusions of this article are included within the article. The raw data can be requested from the corresponding author.

## Abbreviations

Alb: Albumin
ALP: Alkaline phosphatase
ALT: Alanine transaminase
ANOVA: One-way analysis of variance
APTM: 14-O-[(4-Amino-6-hydroxy-pyrimidine-2-yl) thioacetyl] mutilin
AST: Aspartate transaminase
*B. subtilis*: *Bacillus subtilis*
Ca: Calcium
Crea: Creatinine
EDTA: Ethylenediaminetetraacetic acid
Glu: Glucose
GM: Glucose minimal
HCT: Hematocrit
HDL: High density lipoprotein
HGB: Hemoglobin
LDL: Low density lipoprotein
MCH: Mean corpuscular hemoglobin
MCHC: Mean corpuscular haemoglobin concentration
MCV: Mean corpuscular volume
MDR: Multidrug resistant
MRSA: Methicillin-resistant *Staphylococcus aureus*
OECD: Organization for economic cooperation and development
P: Phosphorous
PBS: Phosphate buffer saline
PLT: Platelets
RBC: Red blood cell
*S. aureus*: *Staphylococcus aureus*
SD: Sprague-Dawley
SPF: Specific pathogen free
TB: Total bilirubin
TC: Total cholesterol
TG: Triglycerides
TP: Total protein
UN: Urea nitrogen
WBC: White blood cell
